# The Moderating Effects of Sex on Consequences of Childhood Maltreatment: From Clinical Studies to Animal Models

**DOI:** 10.3389/fnins.2019.01082

**Published:** 2019-10-10

**Authors:** Jordon D. White, Arie Kaffman

**Affiliations:** Department of Psychiatry, Yale School of Medicine, Yale University, New Haven, CT, United States

**Keywords:** sex, childhood maltreatment, early life stress, animal models, limited bedding nesting, maternal separation, deprivation, threat

## Abstract

Stress has pronounced effects on the brain, and thus behavioral outputs. This is particularly true when the stress occurs during vulnerable points in development. A review of the clinical literature regarding the moderating effects of sex on psychopathology in individuals exposed to childhood maltreatment (CM) is complicated by a host of variables that are difficult to quantify and control in clinical settings. As a result, the precise role of sex in moderating the consequences of CM remains elusive. In this review, we explore the rationale for studying this important question and their implications for treatment. We examine this issue using the threat/deprivation conceptual framework and highlight a growing body of work demonstrating important sex differences in human studies and in animal models of early life stress (ELS). The challenges and obstacles for effectively studying this question are reviewed and are followed by recommendations on how to move forward at the clinical and preclinical settings. We hope that this review will help inspire additional studies on this important topic.

## Introduction

Childhood maltreatment (CM) is a heterogenous group of childhood adversities (i.e., subtypes) that include, physical abuse, physical neglect, sexual abuse, emotional abuse, emotional neglect, erratic parenting and severe bullying by peers. Exposure to CM is associated with enormous clinical and economic burden as CM exposure accounts for roughly 50% of all childhood psychiatric disorders in the United States (Green et al., 2010). CM increases the risk for multiple psychopathologies, including depression, anxiety, substance abuse, psychosis, and PTSD (Anda et al., 2006; Kaffman and Meaney, 2007; Nemeroff, 2016; Teicher and Samson, 2016). CM also increases the risk for several medical conditions, such as cardiovascular disease, arthritis, metabolic syndrome, cancer, and generally reduced life expectancy (Kaffman and Meaney, 2007; Teicher and Samson, 2016). Interventions that improve quality of parental care in high-risk children lead to robust and sustained improvement in several behavioral and cognitive outcomes (Olds et al., 1998, 2004a,b; Zeanah et al., 2009; Humphreys et al., 2015), supporting a causal relationship between CM and the presence of behavioral abnormalities later in life. Indeed, CM is now recognized as a significant risk factor for abnormal brain development in industrialized countries (Kaffman and Meaney, 2007; Garner et al., 2012; Nemeroff, 2016; Teicher and Samson, 2016) with an estimated cost of $500 billion annually in the United States alone (Fang et al., 2012).

**FIGURE 1:**
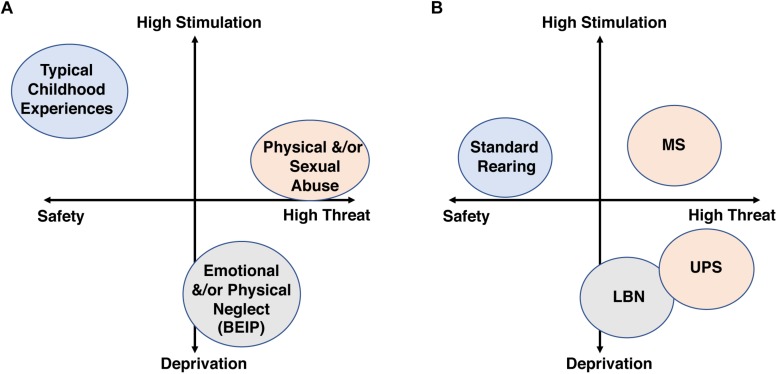
The threat/deprivation conceptualization is shown for human subtypes of CM **(A)** and rodent models of ELS **(B)**.

One of the most robust findings across the CM literature is its additive effect, where the risk for developing a broad range of psychological and medical conditions increases linearly with exposure to a greater number of adversities (Kessler et al., 1997; Anda et al., 2006; Chen et al., 2010; Evans et al., 2013). This dose-dependent effect has led to the development of diagnostic tools that calculate a cumulative-risk score as a way to quantify exposure to CM (Evans et al., 2013). The cumulative model has been expanded by McLaughlin, Sheridan and Lambert who proposed that a two-dimensional “*Threat/Deprivation*” system would better characterize and quantify CM exposure (McLaughlin et al., 2014; McLaughlin and Sheridan, 2016). This model maps adversity along a “*threat*” scale on the *X*-axis and a “*deprivation*” scale on the *Y* axis. Threatening adversities trigger fear of physical harm/death and include experiences that range from physical and sexual abuse to exposure to domestic and neighborhood violence. Deprivation on the other hand is characterized by an early environment that is devoid of appropriate stimulation and parental care and include subtypes such as physical and emotional neglect or severe poverty (see Figure 1A). The authors argue that deprivation and threat lead to different developmental outcomes and psychopathologies. Moreover, they proposed that mapping CM along these two dimensions helps resolve the complexity and heterogeneity of CM allowing for better predicted outcomes when compared to single dimension scale used in the cumulative-risk approach (McLaughlin et al., 2014; McLaughlin and Sheridan, 2016). In this review, we use the *Threat/Deprivation* conceptual model to examine whether CM affects males and females differently in clinical and preclinical studies.

Although a large body of work has shown that CM affects male and females differently, very few findings have been replicated across studies and little information is currently available about the mechanisms by which sex moderates the outcomes of CM (see Supplementary Table S1 for a list of key studies that have examined the effects of sex on psychopathology). This review extends previous discussion on this topic (Gobinath et al., 2014; Bale and Epperson, 2015; Cameron et al., 2017) in three important ways. First, we examine the rationale for studying sex as an important moderator of the consequences of CM and how sex can affect response to treatment (section “Sex as an Important Moderator of Consequences of CM”), an issue that has not received adequate attention to date. Second, we utilize the *Threat/Deprivation* conceptual model to review clinical and preclinical studies that have examined the issue of sex (section “Modereating Effects of Sex in Clinical and Preclinical Studies”). Third, we outline challenges and obstacles that hinder progress and make specific recommendations on how to move forward (section “Challenges and Recommendations”).

## Sex as an Important Moderator of Consequences of CM

Male and female brains, physiology, and immune systems differ in many ways (Gillies and McArthur, 2010; McCarthy, 2015; McCarthy et al., 2015). These differences reflect distinct and specialized roles that males and females play in ensuring reproductive success (Cahill, 2006). In mammals, these differences are first established by increased levels of testosterone during a critical period of development in males. Testosterone is aromatized locally and converted to estrogen leading to several structural and functional differences in the brains of males and females. For example, the anteroventral periventricular nucleus (AVPV) is larger in females, and this sexual dimorphism is considered responsible for establishing a pulsatile pattern of GnRH release in males and a cyclical pattern in females that drives ovulation. These structural differences are established during the postnatal period by a wave of apoptosis in GABAergic neurons in the male AVPV. Structural and functional sexual dimorphic variations that emerge early in development are maintained and extended by different levels of sex hormones produced during reproductive age (i.e., estrogens and progesterone in females and testosterone in males). For excellent reviews on this topic see Gillies and McArthur (2010), McCarthy (2015), McCarthy et al. (2015).

These structural and hormonal alterations are responsible for important differences in the way males and females respond to injury, stress, and medications (Gillies and McArthur, 2010). For example, both male and female mice develop chronic neuropathic pain in response to spared nerve injury. However, the mechanisms responsible for this hypersensitivity to pain differs; it is mediated by the brain’s endogenous immune cells (i.e., microglia), in males but not in females (Sorge et al., 2015). Sex differences in response to environmental insults are well documented in neonates. For example, exposure to hypoxic-ischemic injury in pre-term and full-term babies causes significantly more neurologic damage and long-term disabilities in males compared to females. These sex differences are seen in both humans and rodents and are thought to be mediated by higher rates of apoptosis in male neural stem cells (Hill and Fitch, 2012). Recent genomic work found little overlap in genes that are differentially expressed in men and women diagnosed with major depression across multiple brain regions, including the medial prefrontal cortex (mPFC). Similar sex-specific genomic changes were seen in mice exposed to chronic stress, suggesting that these sex-specific changes are evolutionary conserved across mammalian species (Labonte et al., 2017). Network analysis identified the phosphatase Dusp6 as a central hub in depressed women and overexpression of the transcription factor Emx1 as a central hub in depressed men. Knockdown of Dusp6 in the mPFC combined with subthreshold stress induced a depression-like phenotype in female but not male mice. In contrast, the overexpression of Emx1 induced depression-like behavior in males only. Interestingly, down regulation of Dusp6 in females and upregulation of Emx1 in males led to similar increase in spontaneous firing of glutamatergic neurons in the mPFC (Labonte et al., 2017). Together, these findings suggest that different mechanisms converge in males and females to produce major depression and that some but not all interventions will have sex-specific effects when treating depression. These findings highlight the importance of studying disease mechanism in both sexes as it has critical implications for treatment.

Although many studies show that CM causes different outcomes in males and females, very few of these findings have been replicated across studies and there is little information about the mechanisms underlying the moderating effects of sex on CM outcomes (see Supplementary Table S1 and section “Modereating Effects of Sex in Clinical and Preclinical Studies” below). Moreover, as demonstrated above, even in the presence of similar clinical presentation, the mechanisms responsible for these outcomes might be different in males and females, requiring sex-specific interventions. This is an important consideration given the enormous clinical and economic burden associated with CM.

## Modereating Effects of Sex in Clinical and Preclinical Studies

### Sex as a Moderator of Psychopathology

Over the past 30 years, more than 50 studies, including several systematic reviews and meta-analyses have examined the moderating effects of sex on the psychological consequences of CM in humans (Jumper, 1995; Rind and Tromovitch, 1997; Rind et al., 1998; Paolucci et al., 2001; Gershon et al., 2008; Chen et al., 2010). While some studies found that females are more sensitive to CM (McGee et al., 1997; MacMillan et al., 2001; Lansford et al., 2002; Banyard et al., 2004; Fletcher, 2009; Herringa et al., 2013b), others maintain that males are more sensitive (Hibbard et al., 1990; Garnefski and Diekstra, 1997; McGloin and Widom, 2001; De Bellis and Keshavan, 2003; Bergen et al., 2004; Zeanah et al., 2009; Coohey, 2010; Crozier et al., 2014). A third group of studies proposed a more nuanced and complex relationship between sex and CM, suggesting that the outcome depends on the type of maltreatment, genetic vulnerability, the specific circuit involved, and the developmental stage when the outcomes are assessed (Darves-Bornoz et al., 1998; Gershon et al., 2008; Keyes et al., 2012; Bale and Epperson, 2015; Humphreys et al., 2015; Teicher and Samson, 2016; Gauthier-Duchesne et al., 2017). See also Supplementary Table S1.

Of the systematic reviews, one conducted by Gershon et al. (2008) is particularly helpful as it focuses on more than 30 studies with sufficient power to formally assess sex by ELS interactions in both adulthood and adolescence. Of the 14 studies conducted in adulthood, 50% found no sex differences, 29% reported worse outcomes in females, while 21% found worse clinical outcomes in males. In contrast, of the 19 studies conducted in adolescents, 58% found worse outcomes in males, 30% reported no differences, 5% found mixed effects with females more severely affected in some domains and males more affected in others, and only 5% noted worse outcomes in females. No significant sex by CM interactions were reported by 3 other large meta-analyses (Jumper, 1995; Paolucci et al., 2001; Chen et al., 2010); although, these analyses did not separately assess outcomes in adults and adolescents (Supplementary Table S1).

The assertion that male adolescents are more symptomatic across multiple psychopathologies was challenged by a recent study examining the effects of childhood sexual abuse in a large cohort of adolescents (Gauthier-Duchesne et al., 2017). Their findings indicate a mixed effect, with females being more likely to develop PTSD, males being more likely to develop externalizing disorders, and no sex difference in the vulnerability to internalizing disorders. These findings suggest a more complex moderating effect of sex, but further supports the notion that male and female adolescents respond differently to consequences of sexual abuse. Important sex differences were also demonstrated by Keyes et al. (2012) using a large sample of adults (*n* = 34,653, 52% men) in which physical abuse caused a significant increase in externalizing disorders in males while increasing the rate of internalizing disorders in females (Keyes et al., 2012). Interestingly, in contrast to the work conducted in adolescents, exposure to sexual abuse increased the risk for internalizing and externalizing disorders in both male and female adults (Supplementary Table S1). These findings raise the question as to why physical, but not sexual abuse, is associated with different symptomatology in males and females and highlight the complexity by which sex interacts with different forms of CM. Moreover, as discussed above, one should not assume that comparable levels of symptomatology reflect similar developmental changes or response to treatment in males and females.

#### The Bucharest Early Intervention Project (BEIP) as a Model of Deprivation

The BEIP provides a unique opportunity to examine the moderating effects of sex on a relatively well characterized and homogeneous cohort of children that were exposed to high levels of deprivation, with relatively low-level exposure to threat with respect to the *Threat/Deprivation* model (Figure 1A). In this project, Romanian children, orphaned at birth, were placed in government-run institutions. These orphanages were understaffed and caregivers were insufficiently trained and lacked the necessary resources to provide adequate sensory, emotional and cognitive stimulation for these children (Bos et al., 2011). During the first stage of the project, a large cohort of toddlers (*n* = 104, mean ages 21 months), institutionalized for 6–31 months, was characterized and compared to non-institutionalized age- matched controls (*n* = 66) for developmental milestones. Institutionalized toddlers were physically smaller, showed cognitive delays, and had higher levels of behavioral and emotional problems compared to non-institutionalized controls (Smyke et al., 2007). Importantly, male and female toddlers were similarly, affected at this age (Smyke et al., 2007). Further, male and female institutionalized toddlers showed multiple EEG differences compared to non-institutionalized controls which suggested altered neurodevelopment (Marshall et al., 2004). Together, these findings indicate that early deprivation leads to similar physical, emotional, and cognitive deficits across male and female toddlers.

After the initial characterization, the institutionalized toddlers either stayed at the institution or were adopted into middle-class, Romanian families (*n* = 68 children in each group). The chronically institutionalized group (CIG) and the institutionalized and then adopted group (IAG) were followed over time and compared to an age-matched, never-institutionalized group (NIG). This randomized clinical trial-like setup was used to assess the long-term effects of early deprivation/neglect and adoption on the emotional and cognitive development in a fairly large group of children.

The second assessment was conducted when the children were roughly 4.5 years old; at this time, there was a significant interaction between sex and history of institutionalization (CIG and AIG children grouped together). This interaction was driven by increased internalizing, externalizing and ADHD disorders in institutionalized boys compared to institutionalized girls (Zeanah et al., 2009). Unfortunately, no formal assessment for an interaction between sex and history of institutionalization is available for the third assessment conducted at ages 11–15 (Humphreys et al., 2015). While institutionalized females (CIG and AIG grouped together), but not males, showed higher levels of internalizing symptoms compared to controls, the rates of externalizing disorders and ADHD were similarly, elevated in institutionalized males and females. Interestingly, adoption only reduced levels of externalizing symptoms in boys, and adoption had no effect on the rate of internalizing disorders or ADHD (Humphreys et al., 2015).

In summary, the initial assessment at 2 years of age found no sex differences, the second assessment at age 4.5 found increased psychopathology in males, and the third assessment at ages 11–15 found an increased sensitivity to internalizing disorders in females and equal sensitivity between males and females to externalizing disorders and ADHD. It is unclear if these outcomes reflect true changes in the moderating effects of sex over time, or if the different assessment tools and analyses used at each time point are contributing to these reported differences (Supplementary Table S1). These variable outcomes highlight the difficulties of assessing the moderating effects of sex even in a fairly large and homogeneous group of maltreated children.

### CM by Sex Interaction: Lessons From Imaging Techniques

#### Structural MRI

The use of objective, measurable outcomes such as imaging, EEG, neurocognitive testing, and peripheral biomarkers have provided some of the most robust findings on the moderating effects of sex on the consequences of CM. The best example of this is the consistently documented reduced hippocampal volume in adults exposed to CM (Teicher and Samson, 2016). Both men and women show reduced hippocampal volume, but the effect size in men is significantly more pronounced (Teicher and Samson, 2016). These findings provide some of the most compelling evidence for the existence of sex differences among the long-term consequences of CM. Since reduced hippocampal volume is more consistently found in adults with CM, it would be interesting to determine if these structural changes correspond with more pronounced deficits in hippocampal-mediated tasks in adult males as well. Moreover, most of the structural MRI studies to date have focused on hippocampal changes in individuals exposed to high threat subtypes of CM (Teicher and Samson, 2013) and there is a need to clarify how high levels of deprivation experiences may affect hippocampal volume in males and females.

Reduced corpus callosum size is another consistent finding associated with a history of CM, with male adolescents being more affected than age-matched females (Teicher and Samson, 2016). This is consistent with a meta-analysis by Gershon et al. (2008) and other imaging studies (Herringa et al., 2013a; Crozier et al., 2014; Colich et al., 2017) indicating important sex differences in adolescents exposed to CM.

#### Task-Mediated fMRI

Individuals with a history of CM show increased amygdala activation in response to fearful or angry faces (Teicher and Samson, 2016). This result has been replicated in many clinical studies, with similar results also being reported in rodents (Supplementary Table S2). Since the amygdala plays an important role in detecting and responding to threat, this finding provides a possible explanation for the increased anxiety seen in individuals with a history of CM (Anda et al., 2006; Chen et al., 2010; Nemeroff, 2016; Teicher and Samson, 2016). Exposure to CM increased amygdala activation in children, adolescents and adults, suggesting that inappropriate parental care during a critical period of development, alters the amygdala’s response to threat in a manner that persists into adulthood, reviewed in VanTieghem and Tottenham (2018). This assertion is supported by work showing that the presence of maternal cues reduces amygdala activation in normally developing young children, but not in adolescents (Gee et al., 2014). This phenomenon is called “maternal buffering” and is associated with increased negative connectivity between the prefrontal cortex (PFC) and the amygdala in young children, but not in adolescents, indicating that top-down suppression of amygdala activation becomes independent of maternal cues during adolescence, further supporting the idea of a critical period in development.

Therefore, CM may disrupt the normal maturation of connectivity between the PFC and the amygdala during childhood, leading to abnormal amygdala activation and emotional dysregulation throughout life (Herringa, 2017; VanTieghem and Tottenham, 2018). This assertion is supported by work showing that normally developing children (ages 6–10) displayed positive connectivity between the PFC and the amygdala in response to fearful faces, whereas age-matched children raised in an orphanage showed negative connectivity between the PFC and the amygdala (Gee et al., 2013). These findings led the authors to suggest that early parental deprivation leads to precocious maturation of amygdala-PFC connections which serve to help these children negotiate unfavorable environments (Gee et al., 2013). However, the negative connectivity seen in parentally deprived children was associated with elevated amygdala activation and high levels of anxiety (Gee et al., 2013), a pattern that is qualitatively different than the negative connectivity seen in typically reared adolescents. Thus, rather than precocious maturation along normal developmental trajectory, parental deprivation seems to impair the normal maturation of top-down inhibitory tone between the PFC and the amygdala. As discussed further below, additional work is needed to clarify whether parental neglect/deprivation leads to similar alterations in fronto-limbic connectivity compared to individuals exposed to the high threat form of CM.

To our knowledge, only two studies have used task-mediated fMRI to test the effects of CM and sex on fronto-limbic connectivity (Crozier et al., 2014; Colich et al., 2017). Crozier et al. (2014) used the emotional odd-ball task to assess non-emotional and emotional responses in a group of CM children with a history of physical abuse and neglect (*n* = 29, 55% males, ages 8–16). All CM subjects had positive forensic investigation with the Department of Children and Families (DCF) and were assessed using the Kiddie Schedule for Affective Disorders and Schizophrenia-Present and Lifetime Version (Kaufman et al., 1997). The CM group was compared to an age and sex matched control group (*n* = 45, 42% males) and was found to have lower socioeconomic status, lower IQ, and higher rates of both internalizing and externalizing symptoms (Crozier et al., 2014). No sex differences or interactions between CM and sex were found for IQ, internalizing, externalizing symptoms or performance in the task. However, there were many significant interactions between CM and sex in BOLD signal. For example, in response to fearful faces CM females showed reduced activation of the dorsal medial PFC (dmPFC) while CM males showed increased activation in this region. Additionally, CM males showed increased activation over both CM females and controls in both the calcarine region and the left hippocampus. This is the first study to demonstrate extensive differences in how CM affects the way in which male and female adolescents process fearful faces and it is consistent with a growing body of work showing significant sex differences in the sequela of CM among adolescents (Gershon et al., 2008).

Colich et al. (2017) used the Traumatic Event Screening Inventory for children (TESI-R), to characterize a broad range of adversities (e.g., childhood abuse, neglect, moving homes, and witnessing injury) in a large cohort of children (ages 9–13, males *n* = 59, females *n* = 78). These authors used the implicit emotional-regulation fMRI task and analyzed the effect of sex based on Tanner stage, to control for the earlier sexual maturity in females. In their cohort, higher levels of CM were associated with increased internalizing symptoms in females but not in males after controlling for age. Three brain regions (left vlPFC, right dlPFC/vlPFC, and intracalcarine cortex) showed significant CM X sex interactions, with increased activation in females, but not males. Activation of these three regions in females and the intra-calcarine cortex in males was correlated with internalizing symptoms. Further, higher levels of CM were associated with a stronger negative correlation between the PFC and the amygdala, a finding that was seen in both males and females. However, there was no correlation between the strength of these connections and internalizing symptoms (Colich et al., 2017). Although this study found CM X sex differences in emotional processing, there was little overlap with the findings reported by Crozier et al. (2014). Most notably Crozier et al. (2014) found increased PFC activation in CM males and hypoactivation in females, while (Colich et al., 2017) found increased activation in the PFC of females and little change in males. These differences may be a result of the different tasks utilized (e.g., the emotional odd-ball task vs. the implicit emotional-regulation task) or the subtype of CM experienced; most notably, higher levels of threat are reported in the Crozier cohort. Despite these disparities, both studies highlight important sex differences in how the PFC processes threating faces in maltreated male and female adolescents.

#### Fronto-Limbic Connectivity Using rsfMRI and Tractography

Unlike task mediated fMRI, in which brain connectivity may change depending on the nature of the task, functional or structural connectivity obtained using resting state fMRI (rsfMRI) or diffusion tensor imaging (DTI), do not involve an explicit cognitive task, allowing for a more direct comparison between studies. Jalbrzikowski et al. (2017) have used rsfMRI and DTI to characterize the effects of age and sex on amygdala-PFC connectivity in a large cohort of typically developing adolescents (*n* = 246, ages 10–25, 49% females). Using a longitudinal approach, they found an age-dependent reduction in amygdala-PFC connectivity in this cohort. They replicated these findings using an independent cohort and further substantiated these findings using structural tractography. This refinement process was associated with reduced internalizing symptoms during adolescence with similar outcomes seen in males and females (Jalbrzikowski et al., 2017). This is one of the most rigorous studies to examine how fronto-limbic connectivity matures in typically developing males and females, providing a solid ground to investigate how different types of CM alter this pattern of connectivity.

Several studies have examined the effects of early adversity on amygdala-PFC connectivity using rsfMRI (Supplementary Table S2). Some studies reported reduced connectivity (Herringa et al., 2013b; Birn et al., 2014; Wang et al., 2014), others found no change (Burghy et al., 2012; van der Werff et al., 2013), while others noted increased connectivity (Cisler et al., 2013; Philip et al., 2013; Dean et al., 2014; Nicholson et al., 2015). These conflicting findings are likely due to differences in the composition and severity of CM, the age and sex of the subjects, and additional comorbidities, such as a history of substance abuse and/or depression. For an extensive review on this issue see Herringa (2017), Johnson et al. (2018).

Most studies published to date lack sufficient power to test for CM by sex interactions on fronto-limbic connectivity. The only exception is work by Herringa et al. (2013a) which examined the relationship between CM, levels of internalizing symptoms and resting state connectivity in a cohort of adolescents (*n* = 64, ages 18 ± 0.19, 46% females). CM was assessed using the Childhood Trauma Questionnaire and ranged from 25 to 40, indicating a low to moderate severity. Higher levels of CM, were correlated with higher levels of internalizing symptoms, with females having higher overall levels of internalizing symptoms compared to males (Herringa et al., 2013a). Maltreated females showed reduced functional connectivity between the right amygdala and the vmPFC, an effect not seen in CM males. In contrast, reduced connectivity between the left hippocampus and the vmPFC was seen in both males and females exposed to CM (Herringa et al., 2013a). The authors proposed that this “double hit” in females (e.g., reduced connectivity between the mPFC-amygdala and the mPFC-hippocampus) versus a “single hit” in males (e.g., reduced connectivity between the mPFC-hippocampus), may explain the higher symptomatology seen in females exposed to CM. Since the majority of studies did not find higher levels of internalizing symptoms in maltreated adolescent females (Gershon et al., 2008), additional work is needed to confirm these findings and to clarify whether differences in connectivity are due to sex differences and/or the severity of the internalizing symptoms. Nevertheless, this work is consistent with other studies discussed above showing important sex differences between maltreated male and female adolescents.

### Modeling Early Life Stress in Rodents

We use the term early life stress (ELS) to describe work in rodents that attempts to model aspects of CM, focusing on paradigms that use postnatal stress in order to mimic childhood adversity. Rodents exposed to ELS show many of the developmental and behavioral changes reported in humans, suggesting that work in rodents can clarify important details about how different types of ELS alter neurodevelopment and behavior in males and females (see Supplementary Table S2). Although aspects of the moderating effects of sex on ELS have been reviewed by others in recent years (Gobinath et al., 2014; Lucassen et al., 2017; Walker et al., 2017), the rapid progress in this area warranted an updated reexamination of the issue. Moreover, we examine the preclinical work in regards to the deprivation/threat model and have attempted to link important clinical findings with preclinical studies. This is especially relevant for a growing body of work in rodents that has used human imaging tools such as rsfMRI and high-resolution diffusion MRI (dMRI) to examine the effects of ELS on fronto-limbic connectivity in rodents.

Due to a large number of inconsistent findings across the ELS literature, we searched for paradigms that provided distinct features within the deprivation/threat model and also produced reproducible outcomes in male and female rodents across different labs. Unfortunately, the licking and grooming paradigm developed by Meaney et al. (2002) did not provided enough examples of consistent findings across labs to be included in the current review. Thus, we focused on the low bedding/nesting (LBN) paradigm, a model with a relatively high deprivation score and moderate levels of threat, and the maternal separation (MS) paradigm, a model of moderate/low levels of deprivation and high threat level (Figure 1B). Despite a wealth of literature on these two paradigms from a variety of groups, much of the work was done only in males, with very few studies that document reproducible outcomes in both sexes. As a result, we present several reproducible and clinically relevant findings described only in males, in order to highlight the pressing need to further explore the consequences of these paradigms in females.

#### Limited Bedding/Nesting Paradigms

The LBN paradigm was originally developed by Tallie Baram’s lab in an attempt to model chronic postnatal stress due to impoverished nesting condition and fragmented/erratic maternal care (Walker et al., 2017). In the original, and most commonly implemented version, the dam’s access to nesting materials is severely reduced from PND2 to PND9 and pups are raised on an elevated mesh platform, while the control condition receives standard amounts of bedding and nesting material. The paucity of nesting material models an impoverished, sub-standard rearing condition that leads to fragmented maternal care, characterized by rapid transition in and out of the nest (Rice et al., 2008; Heun-Johnson and Levitt, 2016; Molet et al., 2016a). The exact reason for this fragmented maternal care is unclear, but it might reflect a compensatory foraging mechanism aimed at improving nesting conditions. Rodent pups are fully dependent on the dam for survival (Kuhn and Schanberg, 1998; Kaffman and Meaney, 2007). Therefore the erratic but constant maternal presence in the LBN paradigm is likely to induce a less threatening rearing environment compared to the removal of pups from the nest in the absences of any maternal cues used in the MS paradigm (Figure 1B). Moreover, the limited availability of soft nesting material deprives LBN pups of important sensory/tactile cues during a critical period (Kaffman and Meaney, 2007), as opposed to the ample bedding and nesting material provided in the MS paradigm.

Since newly born pups are unable to regulate their body temperature (Lagerspetz, 1962), LBN pups are also likely to experience mild but chronic hypothermia. Mild hypothermia is a form of deprivation that likely plays an important role in mediating several key developmental abnormalities seen in LBN pups, i.e., stunted growth and elevated corticosterone levels (Lagerspetz, 1962; Anisman et al., 1998; Walker et al., 2017). The observation that female pups appear to be more resilient to the effects of hypothermia (Harshaw and Alberts, 2012) may account for some of the sex-specific outcomes reported in this paradigm; including hippocampal dependent cognitive deficits, altered adult neurogenesis, and changes in reward sensitivity and response to threat (see below for more details).

#### LBN Causes Similar Reduction in Body Weight in Male and Females

One of the most robust findings across the LBN literature is a reduction in body weight, an effect found in both rats and mice, with similar pattern in both sexes. This reduced body weight has been reported to persist into adulthood by some (Maniam et al., 2015a, b; Bath et al., 2016, 2017; Goodwill et al., 2018; Johnson et al., 2018), while others have noted only transient reductions at PND9 that are restored by weaning (Brunson et al., 2005; Rice et al., 2008; Kanatsou et al., 2015; Naninck et al., 2015; Arp et al., 2016; Fuentes et al., 2018). This effect is also seen in studies conducted in humans, where delayed (Smyke et al., 2007) and even stunted growth is seen in individuals exposed to severe CM (Grantham-McGregor et al., 2007). While male and female rodents show similar reductions in weight (Naninck et al., 2015; Arp et al., 2016; Moussaoui et al., 2016; Bath et al., 2017; Goodwill et al., 2018; Johnson et al., 2018), important sex-differences are already present during the early postnatal period (see section “Sex as an Important Moderator of Consequences of CM” above) raising the possibility that somewhat different mechanisms drive the slower growth in male and female pups exposed to LBN.

#### LBN Leads to More Significant Hippocampal Deficits in Male Offspring

Abnormal hippocampal function among LBN-reared offspring is consistently reported. The initial reports indicated that hippocampal impairment associated with LBN emerges only in middle-aged or aged animals (Brunson et al., 2005), and while this is also seen in more contemporary studies (Naninck et al., 2015), recent studies have found deficits much earlier when utilizing the novel object location task (NOLT) instead of novel object recognition or the Morris water maze (Molet et al., 2016b; Bath et al., 2017). For example, deficits in NOLT are seen in male and female LBN-reared mice as early as PND21 (Bath et al., 2017) and in LBN-reared male rats at 2 months (Molet et al., 2016b). These deficits are more significant in males compared to females (Naninck et al., 2015; Bath et al., 2017). Naninck et al. (2015) provided evidence that these sex-specific effects were due to reduced neural stem cell survival in the dentate gyrus of adult male, but not female LBN offspring. These findings are consistent with human literature showing greater reduction in hippocampal size in males exposed to ELS. While reduced volume in the dorsal hippocampus of male LBN offspring has also been found using high resolution MRI (Molet et al., 2016b), females were not included in this study. Thus, it is unclear whether this model recapitulates all sex differences reported in human imaging studies.

#### LBN Increases the Susceptibility to Secondary Stress in Male Offspring

Most studies found no effect of LBN on anxiety in male and female offspring (Brunson et al., 2005; Rice et al., 2008; Naninck et al., 2015; Molet et al., 2016a; Bath et al., 2017; Goodwill et al., 2018; Manzano-Nieves et al., 2018). Two reports found increased anxiety following LBN (Molle et al., 2012; Guadagno et al., 2018) and two others noted mixed effects (Wang et al., 2012; Johnson et al., 2018). The absence of robust changes in anxiety might be due to the short developmental window in which the rodents are exposed to LBN or the relatively mild nature of the stressor. Indeed, van der Kooij et al. (2015) found that exposure to LBN between PND10 and PND17, but not PND2-9, leads to increased anxiety in adult male mice. These findings underscore the important role that timing of exposure plays in modifying developmental and behavioral outcomes later in life.

Work from several groups suggests that a “second hit” might be necessary to unmask underlying changes in anxiety in LBN offspring. For example, adding only 6 episodes of unpredictable maternal separation (UPS) at PND14, 16, 17, 21, 22, and 25 to pups raised under LBN conditions followed by nest disruption leads to a robust increase in anxiety that is not seen in LBN mice that were not separated. Importantly, exposure to UPS increased anxiety in adult male but not female littermates (Johnson et al., 2018). Newborn pups depend on their mother for survival (Kuhn and Schanberg, 1998; Kaffman and Meaney, 2007). Therefore, the UPS paradigm exposes LBN pups to increased levels of threat (Figure 1B), which appears to unmask important sex-differences in anxiety-like behavior. Additionally, single-housing adult LBN animals may also unmask important differences in anxiety and response to threat, as adult LBN-reared male rats that were single-housed showed increased anxiety and greater dendritic arborization in BLA neurons compared to LBN females or controls (Guadagno et al., 2018). Additionally, Arp et al. (2016) found that single housed LBN male mice displayed high levels of freezing during the safety period (tone-off) that was not seen in LBN females or CTL mice.

#### Depression-Like Behaviors in LBN Offspring

Inconsistent findings have been reported for the effects of LBN on depression-like behaviors. For example, studies have found increased helpless behavior in the forced swim test (Cui et al., 2006; Raineki et al., 2012) and reduced sucrose preference (Molet et al., 2016a; Bolton et al., 2017) in males exposed to LBN, but provided no information on female behavior. In contrast, three studies found no effect of LBN on helpless behavior in males (Molet et al., 2016a; Bolton et al., 2018; Goodwill et al., 2018). Bolton et al. (2018), showed that reducing CRF expression in the central amygdala of LBN males reverses anhedonia like behavior, providing a possible mechanism to explain the depressive phenotype documented in LBN males. The only study that examined the effects LBN on depression-like behaviors in both males and females, found increased depression-like behavior in female mice and not male littermates (Goodwill et al., 2018). The Goodwill et al. (2018) work is particularly interesting as, in addition to using standard assays of depression-like behaviors like sucrose preference and forced swim test, home-cage behavior, i.e., locomotor activity and self-grooming, was assessed over 5 days. Such prolonged and unbiased assessment of behavior can identify robust behavioral changes in domains such as self-care and energy levels that map well onto clinical presentation of depression. It is currently unclear if the different outcomes with regard to the effects of LBN on sucrose preference in males are due to differences between C57 mice (Goodwill et al., 2018) and Sprague Dawley rats (Molet et al., 2016a; Bolton et al., 2018) or some other methodological differences between these studies. Further work in both male and female LBN offspring is needed to determine whether the LBN-induced depression phenotype is truly sex-specific and what mechanisms, other than CRF expression may be mediating these changes.

#### LBN Increases CRF Levels and Alters Amygdala Connectivity in Males

Work from several groups provided compelling evidence that LBN increase CRF levels in the hippocampus and that this prolonged exposure to high levels of CRF reduces spine density, dendritic arborization, and contributes to hippocampal-dependent cognitive deficits (Chen et al., 2013). In addition, abnormal expression of CRF in the central nucleus of the amygdala appears to induce an anhedonia-like state in LBN male rats (Bolton et al., 2018). Unfortunately, this work was done exclusively in males and it is currently unclear whether similar alterations in CRF are also seen in females and whether elevated levels of CRF cause similar outcomes in males and females. This is one of many examples, some of which are outlined below, in which outcomes in females have not been explored.

Recent advances in imaging has allowed the use of rsfMRI and DTI in rodents to assess the effect of LBN on functional and structural connectivity in fronto-limbic circuits that include the amygdala, prefrontal cortex, and the hippocampus. Such studies allow for direct comparison between humans and rodents and can help clarify whether variations of ELS cause different alterations in fronto-limbic connectivity and whether sex modulates these effects. Using rsfMRI, Johnson et al. (2018) showed that exposure to UPS, a modified version of the LBN paradigm described in section “LBN Increases the Susceptibility to Secondary Stress in Male Offspring,” leads to increased connectivity between the amygdala and the prefrontal cortex, as well as between the amygdala and the hippocampus in adult male mice. The strength of these connections was highly correlated with anxiety-like behavior providing a possible explanation for the increased anxiety seen in UPS male mice compared to control reared males. In the aforementioned study, UPS did not increase anxiety-like behavior in females, so it would be interesting to know whether similar changes in connectivity are seen in UPS females as well. Similarly, UPS, but not LBN leads to robust increase in anxiety-like behavior (Johnson et al., 2018) raising the question as to whether the higher levels of threat associated with the UPS paradigm (Figure 1B) induce a different pattern of fronto-limbic connectivity when compared to LBN.

Direct comparisons between the effects of LBN and UPS on fronto-limbic connectivity have not been reported yet, but three papers have examined the effects of LBN on fronto-limbic connectivity in male rats (Yan et al., 2017; Bolton et al., 2018; Guadagno et al., 2018). Bolton et al. (2018), found increased structural connectivity between the amygdala and the mPFC in LBN-reared males (Bolton et al., 2018). Yan et al. (2017), used an abbreviated scarcity model in which the limited bedding occurred from PND8-12 to assess the effects of LBN and age (PND45 vs. 60) on functional connectivity between the amygdala and the PFC. Although they did not find a robust LBN effect, there was an age-dependent increase in functional connectivity in control rats that was not seen in LBN rats. This change in trajectory was due to a relatively high connectivity in LBN male adolescents that plateaued in adulthood. These results suggest that LBN causes precocious maturation of fronto-limbic connections in males (Yan et al., 2017), an effect consistent with findings reported in parentally deprived children (see Gee et al., 2013 and section “Task-Mediated fMRI”). Guadagno et al. (2018), used rsMRI to assess functional connectivity between the anterior and posterior BLA and the PFC in PND18 and PND74 rats. They found reduced connectivity between the right anterior BLA and the PFC in both ages but mixed effects, i.e., increased or decreased connectivity between the left anterior BLA and the posterior BLA connections with the PFC. In summary, although differences in connectivity between the amygdala and the PFC were found in LBN-reared adult rats (Supplementary Table S2), no consistent pattern emerged in males, and to our knowledge, no group has yet studied this issue in females.

In summary, LBN represents an ELS model with relatively high deprivation score and moderate levels of threat that is associated with important sex-specific effects on hippocampal function and distinct threat/deprivation profile and long-term consequences when compared to the MS paradigm (Figure 1B, and see also section “Maternal Separation Paradigms” below).

### Maternal Separation Paradigms

Despite the fact that maternal separation (MS), maternal deprivation (MD) and brief maternal separation (BMS, also known as handling) are different postnatal stress paradigms that lead to different outcomes, they are commonly lumped together and discussed interchangeably. Several reviews have previously detailed the rationale for developing MS, BMS, and MD and highlighted important differences in their ability to modulate neurodevelopment, physiology, and behavior (Lehmann and Feldon, 2000; Meaney, 2001; Pryce and Feldon, 2003; Schmidt et al., 2011; Tractenberg et al., 2016).

The term MS is used here to describe a group of procedures in which pups are separated for 1–6 h daily during the first 2–3 weeks of life. Such prolonged separation represents a significant threat to rodent pups that are fully dependent on the dam for survival (Kuhn and Schanberg, 1998; Kaffman and Meaney, 2007). Compared to the LBN paradigm, MS pups experience higher levels of stimulation (Figure 1B) due to exposure to a novel environment, and, in many cases, to increased levels of maternal care after reunification with the dam (Reeb-Sutherland and Tang, 2012; Gobinath et al., 2014; Couto-Pereira et al., 2016).

Maternal separation paradigms allow for high level of flexibility in modifying the complexity of the early life stressor, but this added flexibility is responsible for the development of numerous variations, lack of standardization, and difficulties reproducing developmental outcomes (Lehmann and Feldon, 2000; Tractenberg et al., 2016; Murthy and Gould, 2018). Additionally, even when the paradigm is consistent, strain effects are often present (Millstein et al., 2006; Mehta and Schmauss, 2011; Tractenberg et al., 2016). To identify robust and reproducible outcomes we extracted outcomes specifically associated with MS from two systematic reviews (Loi et al., 2015; Tractenberg et al., 2016) and one meta-analysis (Chen and Jackson, 2016).

The work by Loi et al. (2015) is particularly germane as it specifically explores the effects of different ELS paradigms (e.g., MD, MS, BMS, and LBN) on behavior in male and female rodents. When examining these paradigms together, they note a trend for increased vulnerability in males compared to females in tests for social behavior, cognition, and depression-like behaviors. Yet, when only looking at MS studies utilizing both sexes, Loi et al., 2015 did not find a significant effect of MS on anxiety, depression, or hippocampal-dependent function, in either sex. The few studies that reported a significant main effect of MS, did not present a clear outcome with regard to the moderating effects of sex on MS. The vast majority (i.e., 87%) of the MS studies cited by Loi et al. (2015) used rats with only three studies (13%) conducted in mice. The effects of MS, MD and BDS on behavior in the mouse was systematically reviewed by Tractenberg et al. (2016) and revealed a more consistent pattern. Specifically, when focusing on MS studies, there is a trend for increased depression and anxiety-like behavior across studies, but most studies only used males, and several of the studies that used both males and females did not formally assess for an interaction, making it difficult to determine how sex interacts with MS in the mouse.

Chen and Jackson (2016) conducted the only meta-analysis looking at the effects of MS, MD and BMS on pain sensitivity (Chen and Jackson, 2016). They found a significant reduction in pain sensitivity in rodents exposed to ELS that was due to the effects of BMS and not MS on pain sensitivity. In fact, MS studies found an opposite trend for increased pain sensitivity that did not reach statistical significance. Sex emerged as a significant factor in studies involving MS but not BMS, with males showing greater sensitivity compared to females. MS studies in mice (CD1) showed greater effect size compared to studies conducted in rats consistent with the notion that mice are more sensitive to the consequences of MS. This study demonstrates the utility of meta-analysis to quantify an overall effect size for the different paradigms, identify publication bias, and to reveal a significant effect of sex.

#### MS Alters DNA Methylation and Dopaminergic Development in Males

The challenges of producing consistent behavioral outcomes using the MS paradigm is likely responsible for the paucity of studies describing reproducible cellular and molecular changes in offspring exposed to MS. Nevertheless, two important exceptions are worth noting. First, several groups have found increased levels of DNA methyltransferases (DNMTs), including DNMT1, in the brain of adult male offspring exposed to MS (Anier et al., 2014; Boku et al., 2014; Todkar et al., 2015; Ignacio et al., 2017; Park et al., 2018). This is an important observation because DNMT1 plays a critical role in maintaining DNA methylation and transcription across a large number of promoters in neural stem cells, neurons and glia that regulate circuit development, neuroplasticity, and complex behavior (Guo et al., 2011; Heyward and Sweatt, 2015). This type of epigenetic regulation is now recognized as an important mechanism by which early life stress causes long term changes in gene expression in both animals and humans (Kaffman and Meaney, 2007). Exactly how an increase in DNMTs affects circuits that regulate complex behaviors in adult offspring exposed to MS is not fully elucidated, but work by Boku et al. (2014) has shown that elevated levels of DNMT1 in neural stem cells causes hypermethylation of the retinoic acid receptor (RARα) promoter. This increase in DNA methylation reduced RARα expression and impaired NSC differentiation into neuronal pathway *in vitro* (Boku et al., 2014). DNA methylation also plays a critical role in establishing sex-specific differences early in development (McCarthy, 2016), providing a possible mechanism by which MS may alter developmental trajectory in males and females. As seen with other examples noted above, none of the studies examined the effect of MS on DNMTs expression in females, an issue we suspect will provide important details about whether sex modulates the functional consequences of MS.

Secondly, work by Pena et al. (2017) found that exposing pups to MS from P10-20 causes latent vulnerability to depression that is not seen when pups are separated from P2-12. These different outcomes are due to the ability of MS from P10-20, but not from P2-12, to transiently reduce the expression of the transcription factor OTX2 in the ventral tegmental area (VTA) of male mice. Reduced OTX2 levels during this critical period of development impairs dopaminergic innervation and increases vulnerability to additional stress in adulthood (Pena et al., 2017). It is important to note that the MS paradigm used by Pena et al. (2017) also utilized low amounts of bedding commonly used in the LBN paradigm (i.e., increased levels of deprivation) and required exposure to additional trauma in adulthood in order to induce a depression-like phenotype. Additional studies are needed to clarify whether MS from P10-20 also reduces expression of OTX2 in females and whether OTX2 plays a similar role in dopaminergic development in both sexes.

## Challenges and Recommendations

As discussed above, many questions are yet to be clarified about the moderating effects of sex on consequences of CM and outcomes of ELS in animal models. In this section, we highlight key obstacles for effectively studying this question and make several recommendations about how to overcome these challenges. We first discuss these issues in clinical setting and then examine them in preclinical studies.

### Challenges in Clinical Setting

The conflicting clinical results are not surprising given the number of variables involved and their complex interaction. For instance, different subtypes of CM (e.g., physical abuse vs. emotional neglect) cause somewhat different neurodevelopmental and behavioral outcomes (Keyes et al., 2012; McLaughlin et al., 2014; Teicher and Samson, 2016). These different developmental trajectories are further modified by the timing in which the trauma occurred (Bale and Epperson, 2015; Teicher and Samson, 2016) and the genetic vulnerability of the individual (Caspi et al., 2002, 2003; Klengel et al., 2013). Perhaps most relevant to this review, is work indicating that different forms of CM interact differently with sex (see Supplementary Table S1 and section “Sex as a Moderator of Psychopathology”). Moreover, pure forms of CM are rarely encountered, with most cases of CM characterized by a combination of several subtypes of maltreatment (Kessler et al., 1997; Anda et al., 2006; Keyes et al., 2012). Co-occurring types of CM interact with one another in a manner that is not easy to quantify, but affects the risk for psychopathology (Fergusson et al., 1996; Kessler et al., 1997; Anda et al., 2006).

Sex also appears to moderate the prevalence and the nature of certain forms of CM. Specifically, while men experience lower prevalence of sexual abuse (Cutler and Nolen-Hoeksema, 1991; Coohey, 2010; Gauthier-Duchesne et al., 2017), young males are more likely to experience severe and frequent sexual abuse perpetuated by adolescent males while females are more likely to be abused by adult males (Gauthier-Duchesne et al., 2017). Work by MacMillan et al. (2001) provides a good example of how these differences may influence the interpretation of data with regard to the moderating effects of sex on psychopathology (Supplementary Table S1). For instance, adult women exposed to childhood physical abuse, and to a lesser extent sexual abuse, were more likely than males exposed to the same CM to meet criteria for either depression, substance use disorder, or antisocial behavior (MacMillan et al., 2001). Importantly, 33% of the physically abused women in this study were also sexually abused while only 11% of the men that were physically abused reported sexual abuse (MacMillan et al., 2001). These differences raise the possibility that the increased vulnerability seen in women may be due to more severe trauma and not actual sex differences.

One of the most important contributing factors to the confusion is the lack of a unifying method for characterizing CM. Different diagnostic tools are used to assess CM (Supplementary Table S1), making it practically impossible to compare outcomes across studies or to conduct meaningful meta-analyses. Moreover, most scales use the cumulative-risk model and there is a need to develop tools that diagnose CM along the threat/deprivation dimensions. These critical issues have not received enough attention and we hope that this review will help galvanize an effort to implement a uniformly accepted scale in future studies (see also section “Recommendations for Clinical Work” below).

Another issue that complicates the analysis and interpretation of the data is the use of an appropriate comparison group (Banyard et al., 2004). This is important, as the rates of internalizing disorders are almost twice as high in females (Kilpatrick et al., 2003; Altemus, 2006; Gobinath et al., 2014). This female-specific effect raises the question of whether a direct comparison between males and females is even appropriate. For example, Gold et al. (1999), found no difference in the rates of depression and anxiety between adult men and women exposed to childhood sexual abuse. However, when the rates were normalized to the rates seen in the same sex, non-abused general population, men were found to have higher rates of normalized internalizing disorders compared to women (Gold et al., 1999). While intriguing, these findings were not replicated by Banyard et al. (2004) who reported higher rates of internalizing symptoms in women exposed to sexual abuse, but no sex differences when the rates were normalized to the non-abused same sex general population. Another unresolved methodological question is how to address differences in sexual maturation between males and females. Females enter puberty roughly 18 months before males, suggesting that a comparison should be made based on Tanner phase, and not age *per se* (Colich et al., 2017). This issue is further complicated by extensive work showing that CM accelerates entry into puberty in females, with less clear data available on this issue in males (Cowan and Richardson, 2018).

Cultural norms regarding issues of masculinity, femininity and sexual orientation also influence the moderating effects of sex on the consequences of the traumatic experience (Maikovich-Fong and Jaffee, 2010; Gauthier-Duchesne et al., 2017). These cultural expectations may cause reporting biases that affect rates of psychopathology between males and females (Coohey, 2010). Moreover, it may be more culturally acceptable for boys to act aggressively compared to girls, leading to higher levels of externalizing disorders in boys (Coohey, 2010; Gauthier-Duchesne et al., 2017). For a comprehensive discussion of this important issue see (Rutter et al., 2003).

### Recommendations for Clinical Work

We start by highlighting the need to increase awareness that sex differences matter in terms of the developmental consequences of CM and patient response to treatment. This includes the realization that similar presentation does not necessarily mean similar mechanism, and the interaction between threat, deprivation and sex are likely to be complex and circuit specific. Perhaps the most important and necessary change is the implementation of a uniformly accepted scale to assess CM. Such a scale should be guided by the threat/deprivation conceptual model to better map the complexity and heterogeneity of the CM experiences. This is not a call for the elimination of all other scales, but rather an effort to include one common scale that will allow for better comparisons between studies and help conduct meaningful meta-analyses. In addition, given the broad range of psychopathologies seen after exposure to CM, it would be helpful to include a measurement of global psychopathology in the form of the *p* factor in both males and females. For a detailed review on the p factor and its relationship to CM see Caspi et al. (2014), Ronald (2019). Additional studies using objective measurable outcomes such as imaging, neurocognitive testing, and peripheral markers should provide important details about how different types of CM alter specific circuits in males and females. Such studies should be adequately powered to detect sex differences and will help resolve important discrepancies in imaging studies described above.

#### Challenges Faced by Preclinical Studies

A major issue in the preclinical literature is the paucity of studies that have examined outcomes of ELS in both males and females (Loi et al., 2015; Tractenberg et al., 2016). The large historical bias in male-exclusive studies, i.e., 5 to 1, in neuroscience and biomedical research (Beery and Zucker, 2011) led to the 2015 implementation of an NIH initiative emphasizing the importance of sex as a biological variable (Lee, 2018). While the percentage of studies using both males and female rodents has drastically increased from 17 to 38%, very few of those studies (15–25%) utilized sex as an experimental variable of interest (Beery and Zucker, 2011; Will et al., 2017). Moreover, methodological issues related to statistical analyses and reporting bias have also contributed to the large number of inconsistent findings in the preclinical ELS literature. For example, formal assessment of general linear modeling (GLM) assumptions, e.g., normal distribution and/or equal variance across groups, are often lacking, sample sizes are frequently low without proper justification or power analysis (Button et al., 2013; Dumas-Mallet et al., 2017; Smith, 2017), and the inadequate analysis of “nested” data (Aarts et al., 2014) can all lead to an increased rate of false-positive reporting (Colquhoun, 2014). This is particularly relevant for ELS studies where both fixed (rearing condition) and random (dam) effects are present. In other words, the behavior of each pup is nested within the dam (or litter), thus yielding clustered observations that cannot be considered fully independent, making the traditional use of GLM problematic. This tendency toward underpowered studies and inadequate data analysis may mask individual litters driving effects, making both within group and between group replication more difficult.

Beyond issues related to analysis, it can be difficult to compare findings within the same paradigm, as the specifics of the stress timing, testing age, animal species or strain can directly alter results. For instance, in a systematic review conducted by Tractenberg et al. (2016), MS paradigms were found to be highly varied in terms of separation length, animal strain utilized, and biological and behavioral phenotypes. Variability in methodology is further complicated by inadequate reporting practices. For instance, according to Tractenberg et al. (2016), only half of the 96 studies included in their systematic review met 75% of the criteria for guidelines on reporting animal research, and only three studies had a quality score above 90% (Tractenberg et al., 2016). Additionally, some reports demonstrate little to no strain variation (Millstein and Holmes, 2007), while others show resiliency to MS paradigms in certain strains, e.g., C57bl/6 (Own and Patel, 2013). This inconsistency suggests a need for more in depth methodological reporting or more standardized paradigms between research groups.

An important statistical tool to address conflicting results is to conduct systematic reviews followed by meta-analyses. This approach has been used frequently in clinical studies, but is rarely used to resolve inconsistent findings in preclinical studies in general, and to an even less extent in the ELS literature. In fact, while we are aware of only one meta-analysis that has conducted this kind of analysis using rodent models of ELS (Chen and Jackson, 2016), this type of approach can be very effective in addressing the relative vulnerabilities of males and females to different paradigms of ELS and in identifying important moderators and publication biases.

Finally, preclinical studies would also benefit from using the threat/deprivation conceptual model to study consequences of ELS (Figure 1B). In this regard, one of the most important caveats is that the “standard rearing” condition provides fairly low levels of stimulation that does not adequately reflect the complexity seen in nature or the levels of stimulation seen in children exposed to normal rearing conditions.

#### Recommendations for Preclinical Studies

There is a desperate need for additional work directly exploring outcomes and underlying mechanisms in both males and females exposed to forms of ELS (see sections “Modeling early life stress in rodents” and “Maternal Separation Paradigms”). The use of human imaging modalities such as rsfMRI and dMRI provide a particularly promising area of translational research that can help clarify how sex moderates the effects of deprivation and threat on many aspects of brain development. Such imaging findings should be coupled with genomic, retrograde tracing, optogenetic tools, and behavioral assays to rigorously clarify how these structural and functional changes alter complex behavior in males and females. To improve the clinical relevance of these ELS models, additional enrichment/stimulation during early development in the control group is warranted, and could unmask small, but consistent outcomes that have otherwise been overlooked. Finally, effort should be made to improve the standardization and reporting of rearing conditions and appropriate sample sizes and statistical tools, i.e., hierarchical linear modeling (Woltman et al., 2012; Aarts et al., 2014) should be utilized; see Naninck et al. (2015) for an example of this strategy used in ELS research. Finally, conducting more meta-analyses using animal models of ELS could prove helpful in identifying subtle-sex differences in behavioral and developmental outcomes and help correct for publication bias.

## Conclusion

Important sex differences are present early in development affecting the way males and females respond to environmental challenges early in life. Despite the large number of inconsistent clinical and preclinical findings, a growing body of work has identified important differences in the way sex moderates outcomes of CM. These sex differences will likely have important treatment implications, and, therefore deserve additional research effort to elucidate them. However, such effort would need to address key obstacles at both the clinical and preclinical levels. The most important suggested changes include the development of a uniformly accepted method of characterizing CM and the use of advanced human imaging tools in preclinical studies.

## Author Contributions

Both authors contributed equally to reviewing the literature, conceptualizing the questions, writing and editing the manuscript.

## Conflict of Interest

The authors declare that the research was conducted in the absence of any commercial or financial relationships that could be construed as a potential conflict of interest.
