# Supplementary material for: The Moderating Effects of Sex on Consequences of Childhood Maltreatment: From Clinical Studies to Animal Models
Source: Front Neurosci. 2019 Oct 10;13:1082. doi: 10.3389/fnins.2019.01082 (PMC6797834; doi:10.3389/fnins.2019.01082)
Supplement: Supplementary file 1 [file Table_1.DOCX]

**Table S1.** Inconsistent reports regarding the moderating effects of sex on psychopathology associated with CM. Abbreviations: disorder (d/o), CSA- (childhood sexual abuse), Childhood trauma questionnaire (CTQ), Department of children and families (DCF), chronically institutionalized group (CIG), Institutionalized adopted group (IAG), Never-institutionalized group (NIG).

| Systematic reviews & Meta-Analyses | | | | |
| --- | --- | --- | --- | --- |
| Reference | Sample description | Assessments | Types of maltreatment | Main Findings |
| Jumper 1995 | Meta-analysis of 23 studies, with a total n= 6,878 adult subjects | Depression, self-esteem, psychological symptomatology (e.g. anxiety, somatic d/o, psychosis, etc.) | Sexual abuse | Sexual abuse is associated with increased risk for depression, low self-esteem and psychological symptomatology. No significant sex differences emerged in any of the measurements. |
| Paolucci 2001 | Meta-analysis of 37 studies, with a total n= 25,367 adult subjects | PTSD, suicide, sexual promiscuity, victimization, academic performance | Sexual abuse | Sexual abuse is associated with increased risk for all outcomes measured, with no significant differences between males and females. |
| Gershon 2008 | Systematic review of 30 studies that formally assessed for sex X CM interaction, with separate analyses for adolescents and adults | Multiple psychopathologies including anxiety, depression, and substance abuse. | Sexual abuse, physical abuse, peer victimization | CM is associated with worse outcomes in male adolescents with no clear sex differences in adulthood |
| Chen 2010 | Large meta-analysis using 37 studies (17 case-control studies and 20 cohorts) with a total n= 3,162,318 participants | Anxiety, depression, eating d/o, PTSD, sleep d/o, somatization d/o, schizophrenia and suicide attempts. | Sexual abuse | Sexual abuse increases the risk for anxiety d/o, depression, eating d/o, PTSD, sleep d/o and suicide attempts in both men and women (no interaction between sex and sexual abuse). History of rape was associated with increased risk for psychopathology. |
| Multiple forms of maltreatment- cumulative risk | | | | |
| Reference | Sample description | Assessments | Types of maltreatment | Main Findings |
| Keyes 2012 | General population sample of 34,653 adults living in the USA, 52% men | Internalizing and externalizing dimensions of psychopathology. Used structured interview and a 19-item questionnaire from the Conflict Tactics Scale and the Childhood Trauma Questionnaire. | Sexual abuse, physical abuse, emotional abuse, physical neglect, emotional neglect | Different forms of maltreatment lead to different vulnerabilities to internalizing and externalizing dimensions, with different effects of sex in some but not all forms of abuse. For example, sexual abuse increased risk for both internalizing and externalizing psychopathology in males and females, while physical abuse caused a significant increase in externalizing dimension in males and internalizing dimension in females. |
| MacMillan 2001 | General population sample of 7,016 Ontario Canada residents, ages 15-64, 47% men | Anxiety d/o, major depression, alcohol use d/o, illicit drug abuse/dependence, antisocial personality d/o, and any psychiatric diagnosis. Used diagnostic Interview and The Child Maltreatment History Self-Report. | Physical and sexual abuse | Both physical and sexual abuse increase the risk for many psychiatric d/o and this effect was more pronounced in women across almost all diagnoses. The sample size for sexual abuse in males was underpowered and there is a concern for higher rate of physical/sexual abuse in females compared to males. |
| Kessler 1997 | National comorbidity Survey that include 5877 respondents, ages 15-54, representative of the US population | Assessed the relationship between 26 early adversities and adult psychopathology. | 26 early adversities | CM is more consistently associated with onset but not persistent of psychopathologies. There is an additive nature of adversities of risk for adult psychopathology. Adversities are more likely to co-occur. The effect is not specific with regard to psychopathology. There is no consistent effect of sex. |
| Hibbard 1990 | cohort of adolescents (n= 3998), grades 7^th^-12th | Emotional (internalizing) and behavioral (externalizing) d/o | Physical and/or sexual abuse | Both physical and sexual abuse were associated with increased emotional and behavioral risk, with individuals reporting both sexual and physical abuse showing the highest risk (additive risk). No significant sex X physical abuse interaction were founds. Significant sex by sexual abuse interaction for behavioral but not emotional d/o |
| Arnow 2011 | 5,673 adults (ages 21-71), 57% women, from 31 clinics in North California | Depression; Collected data on CM using CTQ and depression using PHQ8. | Sexual abuse, physical abuse, emotional abuse, physical neglect, emotional neglect | All forms of CM increased rate of depression in a dose dependent manner. There was no significant interaction between any of the maltreatments and sex. Rates of victimization and depression were higher in women. |
| Sexual abuse (high threat) | | | | |
| Reference | Sample description | Assessments | Types of maltreatment | Main Findings |
| Gauthier-Duchesne 2017 | Sample of 447 sexually abused children (319 girls, 128 boys) ages 6-12 | Conducted path analysis to assess the effects of gender, severity of sexual abuse, and guilt on rates of PTSD, internalizing and externalizing d/o | Sexual abuse | Boys experienced more severe and frequent abuse compared to girls. Boys were more likely to be abused by other teenagers while girls were more likely to be abused by adult males. Girls were more likely to likely to develop PTSD. Boys were more likely to develop externalized d/o. No sex differences on the rate of internalizing behavior |
| Banyard 2004 | No CSA males= 37, females= 46, CSA males= 69, females= 128. Sexual abuse occurs at ages 1-14 and interviews conducted at ages 20-30 | Raw and same-sex normalized scores for depression and anxiety | Childhood sexual abuse (CSA) | Exposure to CSA is associated with higher raw scores for anxiety and depression in women compared to men, but these differences were no longer present when normalized to non-abused same sex controls. |
| Coohey 2010 | A group of 158 adolescents (girls= 127, boys = 31), ages 11-14, with substantiated h/o sexual abuse. | Internalizing d/o | Sexual abuse | Boys showed increased rate of internalizing d/o compared to girls |
| Maikovich-Fong 2010 | DCF cases (n=599) of alleged sexual abuse in children-adolescents ages 4-16, 25% males | Internalizing and externalizing psychopathology. Caregivers were administered the Child Behavior Checklist and youths completed the Youth Self-Report questionnaire and the Trauma Symptom Checklist for Children. | Sexual abuse | No differences in rates of externalizing or internalizing symptoms between male and female youth exposed to sexual trauma |
| Fergusson 1996 | Birth cohort of adolescents from New Zealand (n= 1019, ages 16-18 | Depression, anxiety, conduct d/o, alcohol use, suicide. | Sexual abuse | Dose dependent effect of sexual abuse on all psychopathologies tested, with individuals reporting intercourse showing the highest vulnerabilities. Sex was a significant confounder but the dose dependent effect of CSA was still significant after adjusting for sex. |
| Bucharest Early Intervention Project (high deprivation) | | | | |
| Reference | Sample description | Assessments | Types of maltreatment | Main Findings |
| Marshall 2004 | Children from the Bucharest Early Intervention Project (BEIP). Ages 5-31 months from the Institutionalized group (n=104), age matched controls (n=46) | EEG | Parental deprivation/ institutionalization ages 6-31 months | Institutionalized group showed increased low frequency, reduced high frequency, and increased hemispheric asymmetry compared to controls, with no differences between males and females. |
| Zeanah 2009 | Children from the Bucharest Early Intervention Project (BEIP). Age 2.5y, IAG (n= 59), CIG (n= 52), NIG (n= 59). | Externalizing d/o, internalizing d/o and, ADHD using the Preschool Age Psychiatric Assessment (PAPA) administered to the biological parents, foster parents, or institutional care giver and DSM IV for diagnoses | Parental deprivation/ institutionalization ages 6-31 months after birth followed by randomized fostering some of the children. | Exposure to institutionalization caused higher levels of multiple psychopathologies in boys compared to girls. These include externalizing d/o, internalizing d/o and, ADHD. Also, while foster care reduced multiple psychiatric symptoms and impairment in females, it did not reduce psychopathology in males. |
| Humphreys 2015 | Children from the Bucharest Early Intervention Project (BEIP). Age 11-15, IAG (n= 55), CIG (n= 55). NIG (n= 49) | Externalizing d/o, internalizing d/o and, ADHD using the Diagnostic Interview Schedule for Children, 4th edition (DISC-IV) with the biological parents, foster parents, or institutional care giver and DSM IV for diagnosis | Social-parental deprivation/ institutionalization ages 6-31 months after birth followed by randomized fostering some of the children. | Exposure to institutionalization increased internalizing d/o in females but not males, with no sex differences in rates of externalizing d/o or ADHD.  Adoption reduced externalizing behavior in males but not females and had no effect on rates of internalizing d/o or ADHD in males and females. |

**Table S2.** Robust consequences of CM in humans and their parallel findings in paradigms of postnatal stress in rodents.

| Outcome | Human studies/reviews | Rodent studies/reviews |
| --- | --- | --- |
| 1. CM leads to a dose-dependent increase in a large number of psychopathologies and medical conditions | Anda et al., 2006; Chen et al., 2010; Kaffman and Meaney, 2007; Nemeroff, 2016; Teicher and Samson, 2016 | Johnson et al., 2018; Kaffman and Meaney, 2007; Murthy and Gould, 2018; Tractenberg et al., 2016; Walker et al., 2017 |
| 1. Different types of ELS cause different developmental and behavioral outcomes | Keyes et al., 2012; McLaughlin et al., 2014; Teicher and Samson, 2016 | Chen and Jackson, 2016; Johnson et al., 2018; Pena et al., 2017; Pryce and Feldon, 2003 |
| 1. Reduced hippocampal volume in adulthood (M > F) | Teicher and Samson, 2016 | Bath et al., 2017; Naninck et al., 2015; Molet et al., 2016 |
| 1. Abnormal myelination notable for reduced corpus callosum size (M > F) | Teicher and Samson, 2016 | Berrebi et al., 1988; Bordner et al., 2011; Carlyle et al., 2012; Duque et al., 2012 |
| 1. Increased amygdala activation in response to threat | De Bellis and Hooper, 2012; Garrett et al., 2012; Gee et al., 2013; Grant et al., 2011; Maheu et al., 2010; Malter Cohen et al., 2013; Marusak et al., 2015; McCrory et al., 2013; McLaughlin et al., 2015; Suzuki et al., 2014; Tottenham et al., 2011 | Raineki 2012; Malter cohen 2013 |
| 1. Abnormal amygdala connectivity with the PFC and the hippocampus | Birn et al., 2014; Herringa et al., 2013; Wang et al., 2014;Cisler et al., 2013; Dean et al., 2014; Nicholson et al., 2015; Philip et al., 2013 | Bolton et al., 2018; Guadagno et al., 2018; Johnson et al., 2018; Yan et al., 2017 |
| 1. Elevated markers of peripheral immune activation (M = F) | Baumeister et al., 2016; Coelho et al., 2014 | Delpech et al., 2016; Roque et al., 2015 |

**REFERENCES**

Anda, R.F., Felitti, V.J., Bremner, J.D., Walker, J.D., Whitfield, C., Perry, B.D., Dube, S.R., and Giles, W.H. (2006). The enduring effects of abuse and related adverse experiences in childhood. A convergence of evidence from neurobiology and epidemiology. *Eur Arch Psychiatry Clin Neurosci* 256**,** 174-186.

Arnow, B.A., Blasey, C.M., Hunkeler, E.M., Lee, J., and Hayward, C. (2011). Does gender moderate the relationship between childhood maltreatment and adult depression? *Child Maltreat* 16**,** 175-183.

Banyard, V.L., Williams, L.M., and Siegel, J.A. (2004). Childhood sexual abuse: a gender perspective on context and consequences. *Child Maltreat* 9**,** 223-238.

Bath, K.G., Nitenson, A.S., Lichtman, E., Lopez, C., Chen, W., Gallo, M., Goodwill, H., and Manzano-Nieves, G. (2017). Early life stress leads to developmental and sex selective effects on performance in a novel object placement task. *Neurobiology of Stress* 7**,** 57-67.

Baumeister, D., Akhtar, R., Ciufolini, S., Pariante, C.M., and Mondelli, V. (2016). Childhood trauma and adulthood inflammation: a meta-analysis of peripheral C-reactive protein, interleukin-6 and tumour necrosis factor-alpha. *Mol Psychiatry* 21**,** 642-649.

Berrebi, A.S., Fitch, R.H., Ralphe, D.L., Denenberg, J.O., Friedrich, V.L., Jr., and Denenberg, V.H. (1988). Corpus callosum: region-specific effects of sex, early experience and age. *Brain Res* 438**,** 216-224.

Birn, R.M., Patriat, R., Phillips, M.L., Germain, A., and Herringa, R.J. (2014). Childhood maltreatment and combat posttraumatic stress differentially predict fear-related fronto-subcortical connectivity. *Depress Anxiety* 31**,** 880-892.

Bolton, J.L., Molet, J., Regev, L., Chen, Y., Rismanchi, N., Haddad, E., Yang, D.Z., Obenaus, A., and Baram, T.Z. (2018). Anhedonia Following Early-Life Adversity Involves Aberrant Interaction of Reward and Anxiety Circuits and Is Reversed by Partial Silencing of Amygdala Corticotropin-Releasing Hormone Gene. *Biol Psychiatry* 83**,** 137-147.

Bordner, K.A., George, E.D., Carlyle, B.C., Duque, A., Kitchen, R.R., Lam, T.T., Colangelo, C.M., Stone, K.L., Abbott, T.B., Mane, S.M., Nairn, A.C., and Simen, A.A. (2011). Functional genomic and proteomic analysis reveals disruption of myelin-related genes and translation in a mouse model of early life neglect. *Front Psychiatry* 2**,** 18.

Carlyle, B.C., Duque, A., Kitchen, R.R., Bordner, K.A., Coman, D., Doolittle, E., Papademetris, X., Hyder, F., Taylor, J.R., and Simen, A.A. (2012). Maternal separation with early weaning: a rodent model providing novel insights into neglect associated developmental deficits. *Dev Psychopathol* 24**,** 1401-1416.

Chen, L., and Jackson, T. (2016). Early maternal separation and responsiveness to thermal nociception in rodent offspring: A meta-analytic review. *Behav Brain Res* 299**,** 42-50.

Chen, L.P., Murad, M.H., Paras, M.L., Colbenson, K.M., Sattler, A.L., Goranson, E.N., Elamin, M.B., Seime, R.J., Shinozaki, G., Prokop, L.J., and Zirakzadeh, A. (2010). Sexual abuse and lifetime diagnosis of psychiatric disorders: systematic review and meta-analysis. *Mayo Clin Proc* 85**,** 618-629.

Cisler, J.M., James, G.A., Tripathi, S., Mletzko, T., Heim, C., Hu, X.P., Mayberg, H.S., Nemeroff, C.B., and Kilts, C.D. (2013). Differential functional connectivity within an emotion regulation neural network among individuals resilient and susceptible to the depressogenic effects of early life stress. *Psychological Medicine* 43**,** 507-518.

Coelho, R., Viola, T.W., Walss-Bass, C., Brietzke, E., and Grassi-Oliveira, R. (2014). Childhood maltreatment and inflammatory markers: a systematic review. *Acta Psychiatr Scand* 129**,** 180-192.

Coohey, C. (2010). Gender differences in internalizing problems among sexually abused early adolescents. *Child Abuse Negl* 34**,** 856-862.

De Bellis, M.D., and Hooper, S.R. (2012). Neural substrates for processing task-irrelevant emotional distracters in maltreated adolescents with depressive disorders: a pilot study. *J Trauma Stress* 25**,** 198-202.

Dean, A.C., Kohno, M., Hellemann, G., and London, E.D. (2014). Childhood maltreatment and amygdala connectivity in methamphetamine dependence: a pilot study. *Brain and Behavior* 4**,** 867-876.

Delpech, J.C., Wei, L., Hao, J., Yu, X., Madore, C., Butovsky, O., and Kaffman, A. (2016). Early life stress perturbs the maturation of microglia in the developing hippocampus. *Brain Behav Immun*.

Duque, A., Coman, D., Carlyle, B.C., Bordner, K.A., George, E.D., Papademetris, X., Hyder, F., and Simen, A.A. (2012). Neuroanatomical changes in a mouse model of early life neglect. *Brain Struct Funct* 217**,** 459-472.

Fergusson, D.M., Horwood, L.J., and Lynskey, M.T. (1996). Childhood sexual abuse and psychiatric disorder in young adulthood: II. Psychiatric outcomes of childhood sexual abuse. *J Am Acad Child Adolesc Psychiatry* 35**,** 1365-1374.

Garrett, A.S., Carrion, V., Kletter, H., Karchemskiy, A., Weems, C.F., and Reiss, A. (2012). Brain activation to facial expressions in youth with PTSD symptoms. *Depress Anxiety* 29**,** 449-459.

Gauthier-Duchesne, A., Hebert, M., and Daspe, M.E. (2017). Gender as a predictor of posttraumatic stress symptoms and externalizing behavior problems in sexually abused children. *Child Abuse Negl* 64**,** 79-88.

Gee, D.G., Gabard-Durnam, L.J., Flannery, J., Goff, B., Humphreys, K.L., Telzer, E.H., Hare, T.A., Bookheimer, S.Y., and Tottenham, N. (2013). Early developmental emergence of human amygdala-prefrontal connectivity after maternal deprivation. *Proc Natl Acad Sci U S A* 110**,** 15638-15643.

Gershon, A., Minor, K., and Hayward, C. (2008). Gender, victimization, and psychiatric outcomes. *Psychol Med* 38**,** 1377-1391.

Grant, M.M., Cannistraci, C., Hollon, S.D., Gore, J., and Shelton, R. (2011). Childhood trauma history differentiates amygdala response to sad faces within MDD. *J Psychiatr Res* 45**,** 886-895.

Guadagno, A., Wong, T.P., and Walker, C.D. (2018). Morphological and functional changes in the preweaning basolateral amygdala induced by early chronic stress associate with anxiety and fear behavior in adult male, but not female rats. *Prog Neuropsychopharmacol Biol Psychiatry* 81**,** 25-37.

Herringa, R.J., Phillips, M.L., Fournier, J.C., Kronhaus, D.M., and Germain, A. (2013). Childhood and adult trauma both correlate with dorsal anterior cingulate activation to threat in combat veterans. *Psychol Med* 43**,** 1533-1542.

Hibbard, R.A., Ingersoll, G.M., and Orr, D.P. (1990). Behavioral risk, emotional risk, and child abuse among adolescents in a nonclinical setting. *Pediatrics* 86**,** 896-901.

Humphreys, K.L., Gleason, M.M., Drury, S.S., Miron, D., Nelson, C.A., 3rd, Fox, N.A., and Zeanah, C.H. (2015). Effects of institutional rearing and foster care on psychopathology at age 12 years in Romania: follow-up of an open, randomised controlled trial. *Lancet Psychiatry* 2**,** 625-634.

Johnson, F.K., Delpech, J.C., Thompson, G.J., Wei, L., Hao, J., Herman, P., Hyder, F., and Kaffman, A. (2018). Amygdala hyper-connectivity in a mouse model of unpredictable early life stress. *Transl Psychiatry* 8**,** 49.

Jumper, S.A. (1995). A meta-analysis of the relationship of child sexual abuse to adult psychological adjustment. *Child Abuse Negl* 19**,** 715-728.

Kaffman, A., and Meaney, M.J. (2007). Neurodevelopmental sequelae of postnatal maternal care in rodents: clinical and research implications of molecular insights. *J Child Psychol Psychiatry* 48**,** 224-244.

Kessler, R.C., Davis, C.G., and Kendler, K.S. (1997). Childhood adversity and adult psychiatric disorder in the US National Comorbidity Survey. *Psychol Med* 27**,** 1101-1119.

Keyes, K.M., Eaton, N.R., Krueger, R.F., Mclaughlin, K.A., Wall, M.M., Grant, B.F., and Hasin, D.S. (2012). Childhood maltreatment and the structure of common psychiatric disorders. *Br J Psychiatry* 200**,** 107-115.

Macmillan, H.L., Fleming, J.E., Streiner, D.L., Lin, E., Boyle, M.H., Jamieson, E., Duku, E.K., Walsh, C.A., Wong, M.Y., and Beardslee, W.R. (2001). Childhood abuse and lifetime psychopathology in a community sample. *Am J Psychiatry* 158**,** 1878-1883.

Maheu, F.S., Dozier, M., Guyer, A.E., Mandell, D., Peloso, E., Poeth, K., Jenness, J., Lau, J.Y., Ackerman, J.P., Pine, D.S., and Ernst, M. (2010). A preliminary study of medial temporal lobe function in youths with a history of caregiver deprivation and emotional neglect. *Cogn Affect Behav Neurosci* 10**,** 34-49.

Maikovich-Fong, A.K., and Jaffee, S.R. (2010). Sex differences in childhood sexual abuse characteristics and victims' emotional and behavioral problems: findings from a national sample of youth. *Child Abuse Negl* 34**,** 429-437.

Malter Cohen, M., Jing, D., Yang, R.R., Tottenham, N., Lee, F.S., and Casey, B.J. (2013). Early-life stress has persistent effects on amygdala function and development in mice and humans. *Proc Natl Acad Sci U S A* 110**,** 18274-18278.

Marshall, P.J., Fox, N.A., and Bucharest Early Intervention Project Core, G. (2004). A comparison of the electroencephalogram between institutionalized and community children in Romania. *J Cogn Neurosci* 16**,** 1327-1338.

Marusak, H.A., Martin, K.R., Etkin, A., and Thomason, M.E. (2015). Childhood trauma exposure disrupts the automatic regulation of emotional processing. *Neuropsychopharmacology* 40**,** 1250-1258.

Mccrory, E.J., De Brito, S.A., Kelly, P.A., Bird, G., Sebastian, C.L., Mechelli, A., Samuel, S., and Viding, E. (2013). Amygdala activation in maltreated children during pre-attentive emotional processing. *Br J Psychiatry* 202**,** 269-276.

Mclaughlin, K.A., Peverill, M., Gold, A.L., Alves, S., and Sheridan, M.A. (2015). Child Maltreatment and Neural Systems Underlying Emotion Regulation. *J Am Acad Child Adolesc Psychiatry* 54**,** 753-762.

Mclaughlin, K.A., Sheridan, M.A., and Lambert, H.K. (2014). Childhood adversity and neural development: deprivation and threat as distinct dimensions of early experience. *Neurosci Biobehav Rev* 47**,** 578-591.

Molet, J., Maras, P.M., Kinney-Lang, E., Harris, N.G., Rashid, F., Ivy, A.S., Solodkin, A., Obenaus, A., and Baram, T.Z. (2016). MRI uncovers disrupted hippocampal microstructure that underlies memory impairments after early-life adversity. *Hippocampus* 26**,** 1618-1632.

Murthy, S., and Gould, E. (2018). Early Life Stress in Rodents: Animal Models of Illness or Resilience? *Front Behav Neurosci* 12**,** 157.

Naninck, E.F., Hoeijmakers, L., Kakava-Georgiadou, N., Meesters, A., Lazic, S.E., Lucassen, P.J., and Korosi, A. (2015). Chronic early life stress alters developmental and adult neurogenesis and impairs cognitive function in mice. *Hippocampus* 25**,** 309-328.

Nemeroff, C.B. (2016). Paradise Lost: The Neurobiological and Clinical Consequences of Child Abuse and Neglect. *Neuron* 89**,** 892-909.

Nicholson, A.A., Densmore, M., Frewen, P.A., Theberge, J., Neufeld, R.W., Mckinnon, M.C., and Lanius, R.A. (2015). The Dissociative Subtype of Posttraumatic Stress Disorder: Unique Resting-State Functional Connectivity of Basolateral and Centromedial Amygdala Complexes. *Neuropsychopharmacology* 40**,** 2317-2326.

Paolucci, E.O., Genuis, M.L., and Violato, C. (2001). A meta-analysis of the published research on the effects of child sexual abuse. *J Psychol* 135**,** 17-36.

Pena, C.J., Kronman, H.G., Walker, D.M., Cates, H.M., Bagot, R.C., Purushothaman, I., Issler, O., Loh, Y.E., Leong, T., Kiraly, D.D., Goodman, E., Neve, R.L., Shen, L., and Nestler, E.J. (2017). Early life stress confers lifelong stress susceptibility in mice via ventral tegmental area OTX2. *Science* 356**,** 1185-1188.

Philip, N.S., Sweet, L.H., Tyrka, A.R., Price, L.H., Bloom, R.F., and Carpenter, L.L. (2013). Decreased default network connectivity is associated with early life stress in medication-free healthy adults. *Eur Neuropsychopharmacol* 23**,** 24-32.

Pryce, C.R., and Feldon, J. (2003). Long-term neurobehavioural impact of the postnatal environment in rats: manipulations, effects and mediating mechanisms. *Neurosci Biobehav Rev* 27**,** 57-71.

Raineki, C., Cortes, M.R., Belnoue, L., and Sullivan, R.M. (2012). Effects of early-life abuse differ across development: infant social behavior deficits are followed by adolescent depressive-like behaviors mediated by the amygdala. *J Neurosci* 32**,** 7758-7765.

Roque, A., Ochoa-Zarzosa, A., and Torner, L. (2015). Maternal separation activates microglial cells and induces an inflammatory response in the hippocampus of male rat pups, independently of hypothalamic and peripheral cytokine levels. *Brain Behav Immun*.

Suzuki, H., Luby, J.L., Botteron, K.N., Dietrich, R., Mcavoy, M.P., and Barch, D.M. (2014). Early life stress and trauma and enhanced limbic activation to emotionally valenced faces in depressed and healthy children. *J Am Acad Child Adolesc Psychiatry* 53**,** 800-813 e810.

Teicher, M.H., and Samson, J.A. (2016). Annual Research Review: Enduring neurobiological effects of childhood abuse and neglect. *J Child Psychol Psychiatry* 57**,** 241-266.

Tottenham, N., Hare, T.A., Millner, A., Gilhooly, T., Zevin, J.D., and Casey, B.J. (2011). Elevated amygdala response to faces following early deprivation. *Dev Sci* 14**,** 190-204.

Tractenberg, S.G., Levandowski, M.L., De Azeredo, L.A., Orso, R., Roithmann, L.G., Hoffmann, E.S., Brenhouse, H., and Grassi-Oliveira, R. (2016). An overview of maternal separation effects on behavioural outcomes in mice: Evidence from a four-stage methodological systematic review. *Neurosci Biobehav Rev* 68**,** 489-503.

Walker, C.D., Bath, K.G., Joels, M., Korosi, A., Larauche, M., Lucassen, P.J., Morris, M.J., Raineki, C., Roth, T.L., Sullivan, R.M., Tache, Y., and Baram, T.Z. (2017). Chronic early life stress induced by limited bedding and nesting (LBN) material in rodents: critical considerations of methodology, outcomes and translational potential. *Stress* 20**,** 421-448.

Wang, L., Dai, Z., Peng, H., Tan, L., Ding, Y., He, Z., Zhang, Y., Xia, M., Li, Z., Li, W., Cai, Y., Lu, S., Liao, M., Zhang, L., Wu, W., He, Y., and Li, L. (2014). Overlapping and segregated resting-state functional connectivity in patients with major depressive disorder with and without childhood neglect. *Hum Brain Mapp* 35**,** 1154-1166.

Yan, C.G., Rincon-Cortes, M., Raineki, C., Sarro, E., Colcombe, S., Guilfoyle, D.N., Yang, Z., Gerum, S., Biswal, B.B., Milham, M.P., Sullivan, R.M., and Castellanos, F.X. (2017). Aberrant development of intrinsic brain activity in a rat model of caregiver maltreatment of offspring. *Transl Psychiatry* 7**,** e1005.

Zeanah, C.H., Egger, H.L., Smyke, A.T., Nelson, C.A., Fox, N.A., Marshall, P.J., and Guthrie, D. (2009). Institutional rearing and psychiatric disorders in romanian preschool children. *Am J Psychiatry* 166**,** 777-785.
